# Coevolution between Cancer Activities and Food Structure of Human Being from Southwest China

**DOI:** 10.1155/2015/497934

**Published:** 2015-11-01

**Authors:** Yawen Zeng, Juan Du, Xiaoying Pu, Jiazhen Yang, Tao Yang, Shuming Yang, Xiaomeng Yang

**Affiliations:** ^1^Biotechnology and Genetic Resources Institute, Yunnan Academy of Agricultural Sciences, Kunming 650205, China; ^2^Kunming Tiankang Science & Technology Limited Company, Agricultural Biotechnology Key Laboratory of Yunnan Province, Kunming 650223, China

## Abstract

Yunnan and Tibet are the lowest cancer mortality and the largest producer for anticancer crops (brown rice, barley, buckwheat, tea, walnut, mushrooms, and so forth). Shanghai and Jiangsu province in China have the highest mortality of cancers, which are associated with the sharp decline of barley.

## 1. Introduction

Natural products are very popular to combat various physiological threats [1]. Vegetables, fruits, spices, herbs, and beverages have opened up new avenue for the role of phytochemicals in the prevention of human chronic diseases like cancer [2]. Functional foods not only are natural bioactive products with food value and promising cancer prevention and therapy [3], but also prevent diseases, suppress aging, enhance biodefense, bioregulation, and so forth [4]. The biopolymers of edible mushrooms make them very good candidates for formulation of novel functional foods for anticancer and so forth [5]. Greater consumption of fruits and vegetables, as well as whole grain products, appears to lower the risk of multimorbidity [6].

Cancer is becoming the most important public health burden in the world. Its incidence is varying among geographical regions, for example, esophageal cancer high in China, lung cancer in USA, and gallbladder cancer in Chile [7]. Each year 11,844 to 121,442 additional cases of lung cancer, 9,129 to 119,176 cases of bladder cancer, and 10,729 to 110,015 cases of skin cancer worldwide are attributable to inorganic arsenic in food [8]. A diet with higher total diversity may reduce the risk of bladder cancer [9]. The dietary factors are the primary cause of nasopharyngeal cancer [10]. The phytochemicals for anticancer drug design from the green husk of* Juglans regia* L. have gained attention worldwide [11]. *β*-glucans from the cell walls of barley, oat, mushrooms, yeast, seaweeds, algae, and bacteria are essential for new therapeutic strategies against cancer [12].

Southwest China is the only geographical area where functional crop production has significant anticancer effects on humans [13]. Yunnan has not only the largest biodiversity center (higher plants >18,000 species; over 500 cultivated plants and 650 species of wild crops), but also the largest reserves of Al, Pb, Zn, Ti, Sn, Cu, and Ni in China [14] and the lowest incidence and mortality of cancers in China. Modern humans originated from Africa between ~100 and 200 Ka, Asia as early as ~130 Ka, and Northern Eurasia by ~50 Ka [15]. Hunger due to food shortage with climate change is the cause of early humans' evolution from Africa to Asia and later into Eurasia. Hunger was the cause of migration for early humans' evolution; however, disease prevention of early human migrations was associated with food structure from centers of crop origin, but coevolution between anticancer activities and food structure of human based on crop origin center from Southwest China is unclear.

## 2. Southwest Is the Lowest Mortality of Cancers in China

The age-standardized incidence rate for cancers in China during 1998~2007 was 1.8586‰, 2.0205‰ in men and 1.5915‰ in women in urban areas, but in rural areas it was 2.4434‰ and 1.3790‰, respectively [16]. Cancer mortality in China during 2004-2005 was 1.3587‰; cancer mortalities of seven regions of China are stated in Table 1: East China (1.6688‰) > Northeast China (1.3921‰) > North China (1.3126‰) > Central China (1.2426‰) > South China (1.2281‰) > Northwest China (1.1433‰) > Southwest China (0.9471‰) [17] (see Table 1). There are significant differences among East China and five other regions (I, IV, V, VI, or VII) for mortality of major cancer; the age-standardized rates of liver cancer in China and world mortalities were 0.184‰ and 0.180‰, respectively [18]. There are significant differences among South China and six other regions (I, II, III, IV, VI, or VII) for mortality of nasopharyngeal cancer. Nasopharyngeal carcinoma incidence and mortality were obviously higher in South China than in other regions and lowest in North China, and its crude incidence and mortality were 0.0316‰ and 0.0153‰, but world age-standardized incidence and mortality were 0.0244‰ and 0.0118‰, respectively [19].

There are significant differences among Northwest China and six other regions (I, II, III, IV, V, or VI) for mortality of cervical cancer and gastric cancer. Dietary habit was an important factor contributing to gastric cancer, especially regular consumption of fried, grilled, high-salt, high-fat, spicy food and drinking boiled brick tea [20]; Wuwei, a city in Northwest China, has high incidence of gastric cancer, which is because of the carcinogens due to lack of Vc, infection of Hp, atrophic gastritis, and heritage [21].

There are significant differences among Northwest China and three other regions (II, V, or VI) for esophageal cancer (*p* < 0.05); intake of dietary fiber is associated with a reduced risk of esophageal cancer for adults [22], whereas the risk is increased with red meat [23]. Millet exhibits multiple biological activities, including anticancer, antioxidant, immunomodulatory, antifungal, and antihyperglycemia effects [24]; the major genetic split for broomcorn millet divided the accessions into an eastern and a western grouping in Northwestern China [25].

There are significant differences among Southwest China and four other regions (I, II, III, or V) for mortality of breast cancer. Mortality rates increased from Southwest to Northeast and from West to East in China from 1991 to 2011 [26]. There are significant differences among Southwest China and three other regions (I, III, or VII) for mortality of leukemia. There is a significant inverse association between high tea consumption and leukemia risk [27]. The patterns of somatic mutation suggest relevant connections between the functional categories of genes driving acute myeloid leukemia [28]. Xuanwei and Fuyuan of Southwest China have strikingly high incidence of lung cancer, due to food with higher Cd and Ti contents [29]. Southwest China is not only the region with lowest incidence and mortality of cancers in China [17] (see Table 1), but also a geographical area richest in anticancer crop.

## 3. The Lowest Cancer Mortality Associated with Anticancer Crops in Yunnan

Yunnan province in China spans approximately 394,000 Km^2^. It borders Vietnam, Laos, and Myanmar. Kunming is the provincial capital of Yunnan, and its elevation is 1894 m. Yunnan is not only the cradle of human childhood, a transitional region among East Asia continent, South Asian subcontinent, and Indo-China Peninsula, but also a core integration area of Chinese culture, Indian culture, and Mid-south Peninsula culture which all merge with the local culture [30]. Yunnan province is renowned for three kingdoms of plants, animal, and nonferrous metals, parallel evolution of crop adaptation to nonferrous metals, and anticancer foods for human being are mostly cultivated in this region.

Yunnan is not only the region with lowest incidence and mortality of cancers (0.541‰) in China, especially esophageal cancer, gastric cancer, liver cancer, leukemia, female breast cancer, and cervical cancer [17] (see Table 1), but also the largest center of origin and diversity of cultivated crops [13]. The foods/plant extracts of turmeric and Chinese goldthread are more likely to be beneficial against cancer [31]. The cysteine-conjugated metabolites of shogaols are novel dietary colon cancer preventive agents [32]. Consumption of herbal tea is associated with reduced risk of colon cancer, but iced coffee increases rectal cancer risk [33]. Yunnan is not only a core integration area of Vavilov's three centers of crop origin, including Chinese Center, Indian Center, and Central Asiatic Center, but also the largest center of origin and diversity of anticancer crop. Major anticancer food structures for crop are as follows.

### 3.1. Association of Brown Rice with Anticancer Activities

Rice originates from a single domestication 8.2–13.5 Kya in the Southwest China [34]. In 2013, global rice yield was 700.7 million tons, but Chinese rice yield was 203.3 million tons. Yunnan is a region not only presenting great genetic diversity, but also the center of genetic differentiation of* indica* and* japonica* subspecies of Asian cultivated rice; however, pigmented rice with similar wild rice with 2384 accessions accounts for 45.1% of rice landrace in Yunnan [35], but white rice for present day human consumption accounts for 95% rice cultivars in China.* MGN-3* from rice bran may represent a novel adjuvant for the treatment of metastatic breast cancer [36]. The momilactone B in rice bran caused G1 cell cycle arrest and apoptosis in U937 cells, which may be related to anticancer activity [37]. Atractylenolide I might contribute to the anticancer effect of germinated brown rice [38]. The purple rice extract could be developed for functional foods for colon cancer prevention [39]. Human* HepG2* cell apoptosis induced by Methanolic-Payao-Purple rice extracts and vinblastine was mediated through a mitochondrial pathway [40]. *γ*-Oryzanol, proanthocyanidin, and *γ*-tocotrienol in red rice extract may have a potential to serve as food-derived chemotherapeutic agents for cancer patients [41]. Thai purple rice cooked under sterilization could be a potential source of protocatechuic acid exerting high antiproliferative activity [42]. Treatments with peonidin-3-glucoside and cyaniding-3-glucoside from black rice extracts significantly reduced the tumor size and volume* in vivo* [43]. China and India are the world's largest producers of rice, which account for 26% and 20% of all world rice production, respectively. Therefore, Southwest China and North India have the lowest incidence and mortality of cancers associated with origin center of rice, especially pigmented rice.

### 3.2. Association of Barley with Anticancer Activities

Barley is the most important crop among functional foods [13]. Yunnan is the center of second origin for two naked barleys and the largest diversity center in China [14], as well as the largest Chinese producer. All *β*-glucans differ by their length and branching structures, which are considered biological response modifiers with health beneficial effects including anticancer activities [44]. The genotypes of vitamin E 31.5**** *μ*g/g dry weight while being of ascorbic acid equivalent antioxidant capacity 158.1** **mg AEAC/100** **g fresh weight are potential candidates for breeding of barley cultivars with high vitamin E content or antioxidant capacity at harvest, even after storage [45]. The content (mg/kg) of tocotrienols for anticancer activities in barley is higher; that is, barley (910) > rice bran (465) > oat (210) > maize (200) > wheat germ oil (189) > rye (92) [46]. Green barley extract induced preferential antiproliferative and proapoptotic signals within B-lineage leukemia/lymphoma cells [47]. The bioactive compounds in germinated barley and other cereals may reduce the risk of diabetic agents and colon cancer [48]. Protocatechualdehyde present in barley suppressed cell growth and induced apoptosis, which may be a result of deacetylase 2-mediated cyclin D1 suppression [49]. Lunasin present in barley has been observed to prevent skin cancer, which could play an important role in the prevention of cancer in humans [50]. Remarkably high reduction of tumorigenesis and induction of apoptosis in the liver section were achieved in the mouse models with barley-Shochu distillation remnants [51]. Germinated barley foodstuff significantly increased the production of a tumor suppressor gene, which showed promising antineoplastic effects [52].

### 3.3. Association of Buckwheat with Anticancer Activities

Yunnan is not only the center of origins and evolution for buckwheat [13, 14], but also the largest Chinese producer of Tartary buckwheat, which accounts for 60.1% in China. Rutin (2215.5 mg/100 g at 7 days) and quercitrin (2301.0 mg/100 g at 8 days) contents after sowing of buckwheat sprouts were approximately 35 and 65 times higher than those of buckwheat seeds [53]. Yunnan golden buckwheat has unique anticancer effects, and its product, “Weimaining” capsules, is the national second-class anticancer drug [54]. TBWSP31 from Tartary buckwheat water-soluble extracts is a novel antitumor protein and apoptosis inducer [55], and also quercetin from its seeds and bran exhibited the strongest cytotoxic effects against the human hepatoma cell line [56]. Tatariside G may be an effective candidate for chemotherapy against cervical cancer [57].

### 3.4. Association of Tea with Anticancer Activities

After water, tea is the most widely consumed beverage [58]; tea is cultivated in Asia which is producing more than 91% of the world. Green tea and quercetin enhanced the therapeutic effect of docetaxel in castration-resistant prostate cancer cells [59]. Green tea polyphenols have strong antioxidants and the inhibition of 16 cancers [13], such as (−)-epigallocatechin-3-O-gallate, induces apoptosis in acute myeloid leukemia cells [60]. The articles on the association of tea with cancer are 3214 according to PubMed literature database. Yunnan province in China is center of origin for* Camellia sinensis*, which has 35 species and 3 varieties, accounting for 76.6% of* Camellia sinensis* in the world [13, 14, 61]. There are more than 500 compounds identified. Tea in Yunnan has 15 accessions for 35.0%–46.8% polyphenols and 14 accessions for 5% caffeine [61]. Epigallocatechin-gallate in green tea has been shown to have antiproliferative activity in colon cancer cells [62]. Catechins of green tea are flavanols, which have many health related characteristics; they especially lower the cytotoxicity and cost of anticancer treatment, inhibit proliferation of breast cancer cells, and block carcinogenesis [1, 63]. Humans would be able to achieve consistent cancer prevention effects provided there is timely intervention of green tea catechins at appropriate high-dose levels [58]. Green tea and coffee consumption has protective effects on esophageal cancer [64]. Therefore, green tea is the most economic and effective method for anticancer treatment.

### 3.5. Association of Walnut with Anticancer Activities

Yunnan is not only the center of origins and evolution for fruits, which has 66 families, 134 genus, and 499 species [65], but also the Chinese largest producer of walnut (2679,000 ha), which accounts for 50.2% of China in 2013. Walnuts are rich in *ω*-3 fatty acids, tocopherols, *β*-sitosterol, and pedunculagin, which slow down the growth of prostate, colon breast, and renal cancers [66]. The tumor size in mice having walnut in their diet was one-fourth than that of the control diet [67]. The *α*-linolenic acid and *β*-sitosterol from walnut oil decreased proliferation of MCF-7 cells [68]. Changes in the miRNA expression profiles likely affect target gene transcripts involved in pathways of anti-inflammation, antivascularization, antiproliferation, and apoptosis [69]. Walnuts decrease the risk of these chronic diseases (cancer, type 2 diabetes, cardiovascular disease, and visceral obesity via inflammation) [70]. Dietary walnut can reduce cancer growth and development by its anticancer mechanism of suppressing the activation of NF*κ*B [71]. The dihydroxy-3,4′-dimethoxyflavone and regiolone from walnut leaves can induce apoptosis in human breast adenocarcinoma cells [72]. The bioactive compounds in walnut green husks are capable of killing prostate carcinoma cells by inducing apoptosis [11].

### 3.6. Association of Mushrooms with Anticancer Activities

Yunnan is richest in having species of wild mushrooms in China, which accounts for 90.2% in China, and 44.1% in the world. It has 670 species of edible mushrooms, which accounts for 72.4% in China, including boletes (224 species) and edible boletes (144 species) accounting for 57.4% and 72.4% in China, respectively [73]. The export of boletes and* Tricholoma matsutake*, having anticancer activities, from Yunnan province is up to 91,780,000 and 57,380,000 USD in 2011 [13]. Medicinal mushrooms have been used to treat cancer, fungal infections, hypertension, diabetes, inflammation, and renal disorders [74]. The most potent extract identified from* Ganoderma lucidum* inhibited the growth of a gastric cancer cell line by interfering with cellular autophagy and cell cycle [75]. Intake of mushrooms seems to be inversely associated with the epithelial ovarian cancer [76]. Polysaccharides from mushrooms have been widely used in far Asia as antitumor, immunostimulating, antimicrobial, hypocholesterolemic, hypoglycemic, and health-promoting agents [5]. The water extract of* Umbilicaria esculenta* has a great potential to be developed into an anticancer agent that targets telomerase [77].

### 3.7. Association of* Panax notoginseng* with Anticancer Activities

Yunnan is the Chinese largest producer of* Panax notoginseng*, which accounts for 97% of the production of China, and more than 400 products were made from it by 1302 companies in China.* P. notoginseng* is a promising candidate in preventing and treating inflammation-associated colon carcinogenesis [78]. Macroporous resin from the leaves of* P. notoginseng* is enriched with low polarity PPD group saponins of 85% ethanol fraction, which is a new alternative source of anticancer saponins [79]. A new protopanaxadiol-type ginsenoside, 6-O-*β*-d-glucopyranosyl-20-O-*β*-d-glucopyranosyl-20(S)-protopanaxadiol-3-one (1), was isolated from the roots of* P. notoginseng*, which exhibited cytotoxic activity against five human cancer cell lines [80]. An arabinogalactan RN1 from flowers of* Panax notoginseng* had an antiangiogenic effect via BMP2 signaling and could be a potential novel inhibitor of angiogenesis [81]. The major saponins in* P. notoginseng* saponin extract were ginsenosides* Rg1* (31.1%) and* Rb1* (34.4%), which may provide significant natural defense against human colon cancer [82]. In addition, Yunnan is the richest in species of* Amorphophalms knojac* (66.7%) and its largest producer in China. The* konjac* glucomannans are associated with a range of health applications which include decreasing the risk of gut cancer and colon carcinogenesis through reduced toxicity of faecal water and precancerous risk factors of human colon cancer [83].

## 4. Lower Cancer Mortality Associated with Anticancerous Food in Tibet

Tibet in China spans approximately over 1,200,000 Km^2^. It borders Myanmar, Bhutan, and Nepal. Tibetans have been adapted to an altitude exceeding 3,500 m in the early Upper Paleolithic [84]. Tibet is one of the regions with lowest cancer incidence and mortality of cancers (0.643‰) in China, especially colon cancer, lung cancer, nasopharyngeal cancer, and leukemia [17] (see Table 1). Major anticancer food structures are as follows.

### 4.1. Association of Naked Barley with Anticancer Activities

Tibet and its vicinity are not only the centers of domestication of cultivated barley [85, 86], but also the world's largest producer of naked barley for major food. Barley and its products are good sources of antioxidants [87], especially anticancer. The most common anthocyanin in the purple barley is cyanidin 3-glucoside, whereas delphinidin 3-glucoside is the most abundant anthocyanin in the blue and black groups [87]. Himalaya 292 for barley mutant has high contents of *β*-glucan (9.7%) and protein (16.4%), as well as amylose starch (81.6%) [88]. The intrinsic differences of *β*-glucans in barley and other cereals will elicit variable immune and anticancer responses. The molecular mechanisms of *β*-glucan-induced signaling in immune cells are essential for the design of new therapeutic strategies against cancer [12, 89]. *β*-D-glucan from barley regulates breast cancer-relevant gene expression and may be useful for inhibiting endocrine-resistant breast cancer cell proliferation [90]. Whole hulless barley is a functional food that can reduce the prevalence of metabolic syndrome [91].

### 4.2. Association of Milk with Anticancer Activities

Milk consumption is prevalent in daily diets, and its lactase persistence is likely to have an independent origin in Tibetans [92]. The milk protein *α*-casein would provide a more natural and nontoxic approach to the development of novel anticancer therapies [93].

In addition, Guizhou is not only one of the regions of lower cancer incidence and mortality of cancers (0.756‰) in China, but also one of the cradles of human childhood.

## 5. The Highest Cancer Mortality Associated with Food in Some Province of China

Human chronic disease outbreak owes its origin to consumption of brown rice and barley, which were the staple diet of the ancient people, whereas white rice and wheat white flour are now consumed as staple foods of modern people [13]. Yunnan Province is the lowest mortality of cancers (0.541‰) in China [17] or all over the world [94], which associated with the largest production base for barley and tea as well as walnut, and so forth in China. East China includes Shanghai, Jiangsu, Zhejiang, Shandong, Anhui, Fujian, and Jiangxi 7 provinces/city, and it is the highest mortality of cancers (1.6688‰) and the most cancer villages (76) in China, which mainly covers some lower reaches of rivers including the Yellow River, Huaihe River, and Yangtze River and also appears near the Dongting Lake and Poyang Lake; the number of cancer villages in China is in turn East China (76) > Central China (28) > North China (24) = South China (24) > Southwest China (12) > Northwest China (3) = Northeast China (3) [95]. The highest cancer mortality associated with barley dropped in some provinces of China, especially Shanghai, Jiangsu, and Zhejiang. Nanjing (107.3 mg/kg) and Shanghai (95.59 mg/kg) as well as Hanzhou (75.7 mg/kg) cities are the highest soil Pb^2+^ concentrations in 35 cities for China [96]. The lipid-transfer protein from barley grain has an affinity to bind Co^2+^ and Pb^2+^ but has no affinity towards Cd^2+^, Cu^2+^, Zn^2+^, and Cr^3+^ [97]. Barley *β*-glucan is a radioprotective agent, and it can enhance radioprotection in the human hepatoma cell line* HepG2* [98]. Barley with polyphenols possesses many other anticarcinogenic activities, and high epicatechin may be related to a reduced risk of breast cancer [99] and colon cancer. Consumption of lunasin from barley could play an important role in cancer prevention [100], but the barley cultivated areas in China in 1935 (6,380,000 ha) were 5.1 times than that in 2012. Shanghai city in 1986 was 6.0 times barley cultivated more than in 2012; Jiangsu province in 1957 (1,401,400 ha) was 7.5 times than in 2012; however Zhejiang province in 1935 (283800 ha) was 9.5 times cultivated than in 2012.

Shanghai city has not only the highest cancer mortality (2.171‰) in China, especially colon cancer, lung cancer, and female breast cancer [17] (see Table 1) but also has the lowest diversity of cultivated crops, and its elevation is 4.0 m. Colorectal cancer increases by 4.2% annually in Shanghai, which is faster than the average increasing rate of the world [101]. Lunasin from soybean cotyledon and barley, a peptide with 43 amino acid residues, demonstrated chemopreventive and anticancer properties against colon and breast cancers [100, 102]. The consumption of barley rice has certain prevention and adjunctive dietary therapy functions for diabetes mellitus, cardiovascular disease, and cancers [103]. Breast cancer is the most common cancer among women in urban China, such as Shanghai, and so forth; however, soy food consumption is significantly associated with decreased risk of breast cancer and lung cancer [104], but cultivated area of soybean in Shanghai is the lowest in China. Fruit intake is inversely associated with the risk of colorectal cancer [105], but cultivated area of fruits in Shanghai is the lowest in China. The high intake of fruits, vegetables, milk, and eggs may reduce the risk of breast cancer, whereas high animal food intake may increase the risk [106]. Age seems to contribute to increased morbidity and mortality of colorectal carcinoma in Yangpu district of Shanghai, but the mortality of colorectal carcinoma appeared higher than the incidence [107].

Jiangsu province has not only the highest cancer mortality (1.936‰) in China, especially gastric cancer and liver cancer [17] (see Table 1), but also a province of lowest altitude (<50 m) in China. The elevation of Nanjing in Jiangsu province is 15.6 m. The barley with anticancer cultivated areas of Jiangsu province in China in 1957 (1,401,400 ha) was 7.5 times than in 2012. Cancer outbreak owes its origin to consumption of barley of the ancient people, which is replaced by wheat white flour of modern people. Cancer villages of main production regions of Chinese wheat are in turn Anhui (26) > Shangdong (16) > Henan (15) > Jiangsu (14) >Hebei (12) [95]. There are significant correlations between topsoil Pb concentration and gastric cancer, as well as grain Hg concentration with liver cancer in humans [108]. Natural lycopene shows a potential anticancer activity and reduces gastric cancer incidence [109]. The tricin from young green barley leaves on melanin production in B16 melanoma cells inhibits melanin biosynthesis with higher efficacy than arbutin, and it could be used as a whitening agent [110]. The annual average crude incidence and age-standardized incidence by world population were 2.52‰ and 1.79‰, respectively, but Jiangsu being an area with relatively low risk of female breast cancer presented cancer registry areas from 2006 to 2010 as 0.703‰ and 0.481‰, respectively [111].

Zhejiang province has very high cancer mortality (1.727‰) in China, especially for liver cancer [17] (see Table 1). The crude incidence of cancer registered in Zhejiang province in 2009 was 3.202‰; however, age-standardized incidence by Chinese and world standard population was separately 1.6199‰ and 2.0792‰; meanwhile, the crude mortality rate was 1.7697‰ and the age-standardized mortality by Chinese and world standard population were 0.7917‰ and 1.0702‰, respectively [112]. The highest soil concentrations in Zhejiang province were 70.36 mg/kg for Pb, 47.49 mg/kg for Cr, 13.51 mg/kg for As, 0.73 mg/kg for Cd, and 0.67 mg/kg for Hg, while Cd caused the greatest cancer risk [113]. The bioaccumulation of heavy metals in food tubers carries a considerable risk for human cancers [114]. The inhibition of cancer cell viability and apoptosis by protocatechualdehyde in barley leaves may be result of activating transcription factor 3 expression through ERK1/2 and p38-mediated transcriptional activation [115].

## 6. Conclusion and Future Prospects

Chronic disease prevention of early human migrations was associated with food structures from crop origin centers, especially from Asia with four centers of crop origin, which account for 58% in the world population. The early modern human of Southwest China was related to many ancestors of Asians. Southwest China, richest in anticancer crop, not only is the most important evolution base of Asian and anticancer crops, but also has the lowest mortality and incidence of cancers in China. Yunnan, richest in anticancer crops, is the cradle of human childhood and the lowest cancer incidence as well as mortality of cancers (0.541‰) in China, especially esophageal cancer, gastric cancer, liver cancer, leukemia, female breast cancer, and cervical cancer, and also the largest center of origin and diversity of functional crops with anticancer activities (brown rice, barley, buckwheat, tea, walnut, mushrooms,* Panax notoginseng*, Knojac, etc.). Tibet is not only one of the regions of lowest incidence and mortality of cancers (0.643‰) in China, especially colon cancer, lung cancer, nasopharyngeal cancer, and leukemia, but also the largest center of origin and diversity of naked barley for major functional foods. Shanghai and Jiangsu, in China, have the highest mortality of cancers (1.936~2.171‰), which are associated with barley cultivated areas dropped about 6.5 times and 7.5 times, respectively. These results further support that Southwest China (especially Yunnan and Tibet) is the center of lowest mortality of cancers (0.643‰) in China based on coevolution between human's anticancer activities and functional foods from crop origin center.

## Figures and Tables

**Table 1:** Geographical distribution of cancer mortality in China [17].

Province/municipality/region	Major cancer ‰	Esophageal cancer (‰)	Gastric cancer (‰)	Liver cancer (‰)	Colon cancer (‰)	Lung cancer (‰)	Nasopharyngeal cancer (‰)	Leukemia (‰)	Female breast cancer (‰)	Cervical cancer (‰)
Beijing	1.3132	0.0876	0.0944	0.1699	0.1173	0.4009	0.0092	0.0442	0.0956	0.0199
Tianjin	1.2443	0.0670	0.1141	0.1699	0.0782	0.4437	0.0079	0.0602	0.0851	0.0309
Hebei	1.3880	0.2962	0.2827	0.2099	0.0516	0.2854	0.0028	0.0337	0.0547	0.0264
Shanxi	1.4831	0.2439	0.4242	0.1606	0.0672	0.3141	0.0028	0.0470	0.0416	0.0422
Inner Mongolia	1.1343	0.1438	0.1955	0.1795	0.0473	0.2810	0.0051	0.0360	0.0703	0.0172
**I** = **North China**	**1.3126**	**0.1677**	**0.2222**	**0.1780**	**0.0723**	**0.3450**	**0.0056**	**0.0442**	**0.0695**	**0.0273**
Liaoning	1.4498	0.0914	0.2065	0.2237	0.0941	0.4614	0.0046	0.0374	0.0775	0.0162
Jilin	1.1325	0.0271	0.1891	0.3172	0.0478	0.4382	0.0037	0.0303	0.0762	0.0231
Heilongjiang	1.5939	0.0600	0.1724	0.3512	0.0940	0.5101	0.0075	0.0420	0.0784	0.0210
**II** =** Northeast China**	**1.3921**	**0.0595**	**0.1893**	**0.2974**	**0.0786**	**0.4699**	**0.0053**	**0.0366**	**0.0774**	**0.0201**
Shanghai	2.1707	0.1432	0.3008	0.2465	0.2328	0.5151	0.0251	0.0483	0.1218	0.0177
Jiangsu	1.9363	0.2834	0.4452	0.3524	0.0828	0.3695	0.0119	0.0485	0.0574	0.0159
Zhejiang	1.7273	0.1122	0.3377	0.3506	0.1121	0.4184	0.0165	0.0382	0.0728	0.0303
Anhui	1.5738	0.2388	0.4133	0.2688	0.0739	0.2776	0.0136	0.0355	0.0440	0.0327
Fujian	1.4119	0.2736	0.1824	0.3190	0.0781	0.2627	0.0293	0.0411	0.0428	0.0191
Jiangxi	1.1986	0.0505	0.1726	0.2523	0.0945	0.2963	0.0301	0.0409	0.0579	0.0479
Shandong	1.6633	0.1062	0.3478	0.3177	0.0824	0.4565	0.0088	0.0479	0.0795	0.0155
**III** = **East China**	**1.6688**	**0.1726**	**0.3143**	**0.3010**	**0.1081**	**0.3709**	**0.0193**	**0.0429**	**0.0680**	**0.0269**
Henan	1.4440	0.3132	0.3031	0.2759	0.0572	0.2551	0.0044	0.0347	0.0556	0.0273
Hubei	1.2895	0.0893	0.2188	0.3299	0.0786	0.2798	0.0171	0.0312	0.0637	0.0275
Hunan	0.9943	0.0437	0.0977	0.1871	0.0765	0.2514	0.0228	0.0393	0.0617	0.0539
**IV** = **Central China**	**1.2426**	**0.1487**	**0.2065**	**0.2643**	**0.0708**	**0.2621**	**0.0148**	**0.0351**	**0.0603**	**0.0362**
Guangdong	1.2788	0.1441	0.1419	0.3175	0.0965	0.2748	0.0529	0.0359	0.0507	0.0205
Guangxi	1.1717	0.0411	0.1536	0.3377	0.0581	0.2607	0.0492	0.0347	0.0633	0.0282
Hainan	1.2339	0.0299	0.2492	0.2811	0.0857	0.2113	0.0718	0.0279	0.0760	0.0296
**V** = **South China**	**1.2281**	**0.0717**	**0.1816**	**0.3121**	**0.0801**	**0.2489**	**0.0580**	**0.0328**	**0.0633**	**0.0261**
Chongqing	1.4632	0.3004	0.2148	0.3189	0.0714	0.3244	0.0080	0.0322	0.0460	0.0117
Sichuan	1.3322	0.1682	0.2659	0.2890	0.0908	0.2709	0.0143	0.0357	0.0445	0.0174
Guizhou	0.7557	0.0195	0.1130	0.1415	0.0599	0.1693	0.0134	0.0310	0.0320	0.0379
Yunnan	0.5410	0.0139	0.0829	0.1023	0.0487	0.0996	0.0077	0.0320	0.0288	0.0167
Tibet	0.6433	0.0386	0.2830	0.2070	0.0077	0.0309	0.0026	0.0026	0.0299	0.0349
**VI** = **Southwest China**	**0.9471**	**0.1081**	**0.1919**	**0.2117**	**0.0557**	**0.1790**	**0.0092**	**0.0267**	**0.0362**	**0.0237**
Shanxi	1.1652	0.1339	0.2836	0.2038	0.0383	0.1875	0.0059	0.0363	0.0542	0.0468
Gansu	1.2771	0.1572	0.4420	0.1946	0.0524	0.1568	0.0030	0.0444	0.0463	0.0756
Qinghai	1.2303	0.1156	0.3232	0.1982	0.0354	0.1994	0.0000	0.0507	0.0412	0.0291
Ningxia	1.1674	0.1008	0.2233	0.2268	0.0679	0.2345	0.0028	0.0462	0.0509	0.0382
Xinjiang	0.8764	0.1941	0.1950	0.1041	0.0201	0.1005	0.0036	0.0379	0.0326	0.0889
**VII** = **Northwest China**	**1.1433**	**0.1403**	**0.2934**	**0.1855**	**0.0428**	**0.1757**	**0.0031**	**0.0431**	**0.0450**	**0.0557**
**China**	**1.3587**	**0.1521**	**0.2471**	**0.2626**	**0.0752**	**0.3083**	**0.0146**	**0.0385**	**0.0590**	**0.0280**

## Abbreviations

ALA: Alpha-linolenic acid
*EPIYA*: Glu-Pro-Ile-Tyr-Ala
*HepG2*: Liver hepatocellular cells
Hp: Haptoglobin
Ka: 1000 years
Km^2^: Square kilometres
Kya: Thousand years ago
MCF-7 cells: Human breast adenocarcinoma cell line
MGN-3: Arabinoxylan rice bran
NF*κ*B: Nuclear factor kappa-light-chain-enhancer of activated B cells
SNPs: Single nucleotide polymorphisms
TBWSP31: Tartary Buckwheat Protein Fraction
USA: United States of America
USD: United States Dollar
Vc: Vitamin C.
